# ‘It’s hard to feel this way every single day’: patients’ perspectives on the emotional burden of narcolepsy

**DOI:** 10.3389/fpsyg.2025.1611500

**Published:** 2025-11-13

**Authors:** Jan Hlodak, Andrea Madarasova Geckova, Zuzana Dankulincova Veselska, Lucia Bosakova, Eva Feketeova

**Affiliations:** 1 Department of Health Psychology and Research Methodology, Faculty of Medicine, Pavol Jozef Safarik University in Kosice, Košice, Slovakia; 2 Olomouc University Social Health Institute, Palacky University in Olomouc, Olomouc, Czechia; 3 Louis Pasteur University Hospital, Košice, Slovakia; 4 Department of Neurology, Faculty of Medicine, Pavol Jozef Safarik University in Kosice, Košice, Slovakia

**Keywords:** narcolepsy, patients’ perspective, qualitative research, narrative semi-structured interviews, emotional burden

## Abstract

**Background:**

Narcolepsy is a chronic neurological disorder characterized by excessive daytime sleepiness and, in the case of narcolepsy type 1, cataplexy. Patients may also experience various sleep disturbances, including hallucinations, sleep paralysis, and vivid dreaming. Beyond its clinical symptoms, narcolepsy impacts aspects of daily life, social functioning, emotional well-being, and overall quality of life. Despite the substantial burden associated with the disorder, qualitative research exploring the emotional burden of individuals with narcolepsy is limited.

**Methods:**

A qualitative study was conducted using narrative semi-structured interviews with 25 individuals diagnosed with narcolepsy. The interview framework was validated by a multidisciplinary panel of experts to ensure methodological rigor. Interviews were conducted either in the patients’ homes or hospital settings, transcribed verbatim, and then analyzed inductively to develop a comprehensive codebook. A thematic analysis was then performed to identify key patterns and insights into the lived experiences of individuals with narcolepsy.

**Results:**

In-depth patient interviews revealed key themes related to the emotional burden of narcolepsy. The onset of symptoms was identified as particularly distressing, evoking feelings of shame, embarrassment, and fear. Symptom manifestation also affected self-perception and important roles in life. Additionally, patients’ experiences of daily life, along with their future aspirations and expectations, contributed to a comprehensive understanding of the challenges associated with narcolepsy.

**Conclusion:**

The impact of narcolepsy is linked with the emotional experiences and self-perception of patients, influencing their quality of life. This burden may also contribute to potential diagnostic delays and hinder the timely initiation of treatment.

## Introduction

Narcolepsy is a chronic neurological disorder characterized primarily by excessive daytime sleepiness (EDS), which significantly impairs daily functioning and quality of life (American Academy of Sleep Medicine, 2014; Sateia, 2014). It is classified as a disorder of central hypersomnolence and results from the irreversible loss of hypocretin-producing neurons in the hypothalamus, leading to dysregulation of the sleep–wake cycle (Barateau et al., 2016). While EDS is a universal symptom among individuals with narcolepsy, only those diagnosed with narcolepsy type 1 (NT1) experience cataplexy—an emotionally triggered, transient loss of muscle tone. Cataplexy can manifest as either partial muscle weakness, affecting specific regions such as the arms, legs, or jaw, or as a complete loss of muscle tone, wherein voluntary movement ceases except for the preservation of ocular and respiratory function (Zhang et al., 2020). This phenomenon is predominantly induced by sudden, often positive, emotional stimuli.

In addition to EDS and cataplexy, many individuals with narcolepsy exhibit symptoms indicative of sleep fragmentation, including hypnagogic and hypnopompic hallucinations, sleep paralysis, and vivid or distressing dreams. Furthermore, cognitive impairments, such as deficits in attention, memory, and executive function, along with persistent daytime sleepiness or fatigue, are frequently reported (Baker et al., 2020). Patients diagnosed with narcolepsy type 2 (NT2) present with a clinical profile similar to NT1 but lack cataplexy (Barateau et al., 2016). According to Hlodak et al. (2025b) differences may also be found in how patients perceive symptom manifestation and overall experiences with the disease, mostly focusing on symptoms onset, influencing factors, and changes in time. Comorbidities may also be present in patients with narcolepsy. Feketeova et al. (2020) describes the comorbidities of Slovak narcolepsy patients: 16% suffer from hypertension, 8% with ischemic heart disease, 18% with dyslipidaemia, 10% with diabetes mellitus type 2, 20% with autoimmune disorders (e.g., Hashimoto thyroiditis), 15% with headache, and 20% with mental disorders. Based on their findings, the presence of narcolepsy doubled the presence of mental disorders (e.g., stress-related, or affective disorders).

The symptomatology of narcolepsy can profoundly affect health-related quality of life (HRQoL) and overall well-being in individuals diagnosed with both NT1 and NT2. The burden of narcolepsy extends beyond its natural manifestations, influencing emotional well-being, social functioning, and daily life management (Hlodak et al., 2025a).

According to Kapella et al. (2015), the presence of EDS and cataplexy significantly contribute to perceived stigmatization, which in turn affects social participation, lowers mood, and reduces overall HRQoL. Moreover, patients with narcolepsy frequently exhibit elevated levels of anxiety and depression. According to Morse and Sanjeev (2018), over half of individuals with narcolepsy experience symptoms of depression and/or anxiety. These findings are further supported by Ruoff et al. (2017), who identified increased rates of psychiatric comorbidities and anxiety levels in narcolepsy patients compared to the general population.

Despite the well-defined symptomatology of narcolepsy, misdiagnosis remains prevalent, often resulting in significant diagnostic delays. A European Narcolepsy Network study reported a mean diagnostic delay of 9.7 ± 11.5 years, highlighting the substantial gap between symptom onset and formal diagnosis (Zhang et al., 2022). This delay underscores the necessity for increased clinical awareness and improved diagnostic strategies to mitigate the long-term psychosocial and functional consequences of the disorder.

Thorpy and Krieger (2014) emphasize that delayed diagnosis results in postponed treatment initiation, prolonging symptom burden and impairing long-term disease management. Additionally, prolonged or uncertain diagnostics has an impact on the social and emotional aspects of the disorder, as patients are frequently mislabeled as lazy, unmotivated, or disinterested by both family members and society in general. Misdiagnosis or diagnostic delays can further contribute to impairments in productivity, academic achievement, and occupational performance (Maski et al., 2017).

To assess the impact of narcolepsy on HRQoL, numerous studies have employed quantitative methodologies, typically comparing HRQoL components between narcolepsy patients and healthy controls (Tadrous et al., 2021). However, despite the growing body of literature, qualitative research exploring firsthand experiences of individuals with narcolepsy remains relatively scarce. Notable exceptions include Schockman et al. (2024), who examined the symptomatology and needs of patients; Franceschini et al. (2020), who investigated experiences with cataplexy; and Tadrous et al. (2024), who explored attitudes toward physical activity among individuals with narcolepsy.

Recognizing the critical role of patient perspectives in understanding the full impact of the disorder, the present study adopted a qualitative approach aimed at capturing the emotional burden of narcolepsy. By conducting in-depth interviews with a sample of maximum heterogeneity of Slovak narcolepsy patients, we aimed to provide a patient-centered understanding of how this condition influences emotional well-being, daily life, and self-perception.

## Methods

### Design and setting

This study is in line with the cross-sectional qualitative narrative research methodology created by the Health Experience Research Group (HERG) at Oxford University focusing on individual patients’ experiences (Ziebland and McPherson, 2006; Ziebland and Herxheimer, 2008; Ziebland and Wyke, 2012). It is a part of a broader research project examining the perspectives of narcolepsy patients, particularly their experiences of the disease and the challenges associated with this condition, using narrative semi-structured interviews (Hlodak et al., 2025b). This study received approval from the Ethics Committee at the Faculty of Medicine of the Pavol Jozef Safarik University in Kosice, Slovakia, and the Ethics Committee of Louis Pasteur University Hospital in Kosice, Slovakia (Quality of life in patients suffering from narcolepsy, 2022–29N/2022), and is in accordance with the 1964 Declaration of Helsinki and its later amendments or comparable ethical standards.

### Procedure

First, we developed an interview guide with the questions and topics for the narrative semi-structured interview, based on a comprehensive literature review. This was further supplemented and approved by a multidisciplinary board consisting of a psychiatrist, psychologists, qualitative researchers, a neurologist, a somnologist, and narcolepsy patients. This approach ensured that every possible aspect of experience related to narcolepsy, including relevant HRQoL domains, was thoroughly addressed. Second, we set the recruitment strategy and started the recruitment process. Third, we interviewed patients and analyzed the data.

### Recruitment and sample

We used purposive sampling to recruit patients using the patient database of the Sleep Laboratory at the Neurology Clinic of L. Pasteur University Hospital in Kosice (sleep clinic). The selection process aimed to achieve the maximum variation within the sample (based on sex, age, education, geographical region, living conditions, marital status, employment status, etc.). Inclusion criteria were: (1) age 18 years or older, (2) proficiency in the Slovak language and (3) diagnosis of narcolepsy type 1 or type 2 according to the International Classification of Sleep Disorders (ICSD) 2nd or 3rd edition criteria (American Academy of Sleep Medicine, 2014; Sateia, 2014), depending on whether a patient was diagnosed before or after the release of ICSD-3. Potential participants were approached by the neurologist (EF) from the sleep clinic. They received an informational pamphlet outlining the purpose and objectives of the study. We subsequently contacted those interested in participating in the study individually – by phone, email, or in person at the sleep clinic – to ensure they understood the purpose of the study, the voluntary nature of their participation, and to address any questions or concerns. After receiving the information, the volunteer patients signed an informed consent form and willingly agreed to participate.

### Interview process

The interviews lasted from 1 to 2 h. Each interview began with an open-ended narrative question: *“Try to recall when you or your close ones first noticed something was wrong.”* This prompt encouraged participants to narrate their personal experiences with the disease. Following the initial narrative, the interview proceeded with a semi-structured format.

Interviews were conducted by the first author (JH), a psychologist trained in qualitative interviewing, at participants’ homes or at the sleep clinic. Each interview was audio-recorded for transcription purposes. Additionally, a second recording (either audio or video) was made based on the participant’s preference, to be used in case of possible issues with the first recording. Participants provided written consent regarding the format of the recordings, specifying whether they permitted their data to be used for patients’ perspectives website development, educational purposes, or research publications. They could also choose between video, audio, or anonymous transcript-based representation for dissemination. Each interview was transcribed verbatim, and participants had the opportunity to review and modify their transcripts by excluding specific segments or altering names before data analysis. Data collection took place from May 2023 to August 2024.

### Data analysis

We systematically reviewed the transcribed interviews and removed all identifying details to maintain anonymity. Initially, we coded the first five interviews inductively to identify emerging themes. After consensual development of a comprehensive codebook within the research team, we integrated additional codes. Using this finalized codebook, we then analyzed all the interviews (including the initial five) deductively with the aid of the MAXQDA software for MAC (v.24), which facilitated the coding process and segment classification. As a result, we applied an abductive approach to narrative analysis. The abductive analytical approach offers several advantages, including broadening the existing understanding of the data, deepening insights from the interviews, enhancing the accuracy and nuance of the analysis, and providing well-grounded explanations for observed phenomena (Hurley et al., 2021). Furthermore, the narrative analysis approach enabled us to identify emerging themes and new insights, contributing to a richer interpretation of the results (Ross and Green, 2011). Three authors (JH, AMG, ZD) independently coded each interview and then engaged in a collaborative, consensus-based coding process for each transcript.

After the initial coding of all interviews, we continued with thematic analysis of specific segments focused on emotional experience of narcolepsy symptoms in line with the purpose of this study. We conducted a thematic analysis (Clarke and Braun, 2017) using the One Sheet of Paper (OSOP) approach (Ziebland and McPherson, 2006), for which all researchers received training. This method involved systematically reviewing each coded segment and documenting all key themes on a single sheet of paper to create a structured thematic overview. We then created a thematic map to illustrate the key themes, subthemes, and their interconnections. Summaries of the findings reflect the thematic maps, highlighting both robust subthemes and less frequently mentioned nuances.

## Results

A total of 25 patients were interviewed and included in this study. Most of the participants were female (72%) and patients with NT1 (92%). This distribution reflects the national prevalence of narcolepsy subtypes in Slovakia, where approximately 84% of narcolepsy patients are diagnosed with NT1 (Feketeova et al., 2020). Most interviewees were young or middle-aged adults, with two 20-year-old patients and two elderly individuals also included. One participant had been diagnosed only a few days prior to the interview. The majority (96%) were Caucasian; one participant was a member of a minority. Eleven participants (44%) were drug-naïve at the time of an interview. The sample reflects the sociodemographic characteristics of the narcolepsy population in Slovakia (Feketeova et al., 2020). A detailed description of the sample is provided in Table 1.

**Table 1:** Sociodemographic characteristics of the sample (*n* = 25).

Sociodemographic specification of sample (*n* = 25) indicator		Prevalence *n* (%)
Narcolepsy type	NT1 NT2	23 (92) 2 (8)
Sex	Male	7 (28)
	Female	18 (72)
Age	Min-Max range	19–77
	Mean [SD]	40.04 [14.97]
Date of Birth	<1950	1 (4)
	1951–1960	1 (4)
	1961–1970	3 (12)
	1971–1980	5 (20)
	1981–1990	5 (20)
	1991–2000	8 (32)
	>2000	2 (8)
Relationship status	Single	9 (36)
	In a relationship	3 (12)
	Married	10 (40)
	Divorced	3 (12)
Have children	Yes	13 (52)
	No	12 (48)
Employment status	Student	2 (8)
	Employed	10 (40)
	Unemployed and w/o pension	2 (8)
	Disability pensioner or retired Pensioner or retired	9 (36) 1 (4)
	Maternity leave	1 (4)
Living location*	Village (<5,000)	8 (32)
	Town (<45,000)	9 (36)
	City (>45,000)	8 (32)

^*^According to the location classification determined by the Municipal Organization Act or the population size.

The interviews were analyzed to explore the emotional burden of narcolepsy. We identified several main topics focused on (a) experiences with the disease onset, (b) feelings of embarrassment, (c) feelings of fear and concerns, (d) fulfillment of self-perception and life roles, (e) emotional experiences of living with narcolepsy, and (f) future aspirations and expectations.

### Experiences with the disease onset

The initial phase of living with narcolepsy was particularly challenging for the study participants, as they must adapt their daily routines to accommodate the symptoms of the disorder, accept various limitations to activities, and navigate social reactions. During this period, EDS and cataplexy are often at their most pronounced, making symptom management especially difficult.

It was a long-term struggle, you know, when you constantly feel sleepy, like you always want to sleep. So, I had to adjust and try to stay as active as possible keeping myself moving and occupied to prevent falling asleep. The worst part for me was sitting somewhere for a long time because that’s when the sleepiness would hit me the hardest, and I couldn’t really suppress it. People eventually started noticing that something was wrong with me, but I had no way of controlling it. I could try pinching myself or something, but that wouldn’t really wake me up completely. So, overall, it was a real struggle.” *Female, 54-years-old, diagnosed with narcolepsy type one (F54NT1a)*

For instance, some participants recalled that this stage was particularly distressing due to the uncontrollable manifestations of cataplexy. Younger participants described this period as emotionally burdening, primarily because of the ridicule and lack of understanding from their peers at school. One patient metaphorically linked the experience to being repeatedly “struck by life,” leaving him feeling disoriented and unable to regain control. The disease onset also significantly elevated anxiety about the diagnostic process, as patients struggled to envision what the process would entail. This was partially caused by a lack of information about narcolepsy and sleep-related problems in the general population. For some participants, the diagnosis was a surprise, considering they never experienced more serious health problems. For those, the diagnoses came as a shock, understanding narcolepsy is untreatable and for life.

“I was definitely surprised…because I had never been sick before…so…that it’s more serious, that it’s something that will stay with me forever…for the rest of my life, yeah, like…maybe the flu or a cold but this…no, this is different, actually I was very surprised…nothing like this had ever affected me before.” *(F22NT1)*

“You know what, at first, I was like, I have to pick up all the illnesses along the way whenever I go somewhere because I already had those cardiological problems before, and then narcolepsy was added to it, so I’m like, wow, I’m really unlucky. I have to collect everything along the way. Some people are perfectly healthy, and I have almost all the diagnoses.” *(F54NT1b)*

Patients in our study frequently discussed ways to come to terms with narcolepsy. Some emphasized the importance of recognizing that narcolepsy is not a life-threatening condition and that there are significantly more severe diseases that can affect individuals. Many found reassurance in knowing that other patients experience similar or identical symptoms and their associated impacts, as well as in the realization that some individuals struggle with the condition more than they do. One male patient initially feared that his symptoms could be indicative of multiple sclerosis, a condition present in his family, which has a progressive nature and the potential to impact his life far more dramatically than narcolepsy; he felt relieved that this was not the case.

“And when I read on forums about the issues narcoleptics face, how they require two or even three different medications and still struggle—some only having about four decent hours in a day—I keep telling myself that, in this context, I am actually doing quite well. And maybe that’s something that helps me maintain a sense of gratitude or optimism, knowing that I have my situation relatively well managed.” *(M37NT1)*

“In the beginning, life is difficult, but even now, it remains challenging. It’s not really living—it’s merely surviving. However, over time, you learn to live with it. By setting your own rules and priorities, you adapt, much like anyone else with a chronic condition. Eventually, you come to realize that others have it even worse. Yes, you may not get much out of life, but you do your best within your limits, trying not to be pressured by time constraints.” *(F52NT1)*

### Feelings of embarrassment

The inability to control narcoleptic symptoms in public settings often placed patients in distressing situations. Many found it particularly troubling when bystanders misinterpreted their sudden sleep episodes as intoxication or treated them as a source of amusement. Cataplexy was frequently mistaken for fainting or an epileptic seizure, drawing immediate and often unwanted attention to the patient. In some cases, well-meaning but uninformed individuals have even called emergency medical services.

Such experiences can lead to profound feelings of embarrassment and humiliation. As a result, some individuals with narcolepsy reduced their public activities or withdrew from workplace interactions to avoid similar incidents.

“It’s hard to feel this way every single day. If only there were just one day where I didn’t feel the weight of people’s stares, where I could say, ‘This was a good day.’ But I don’t have that day—I never do. Every morning, I wake up knowing that I’ll be useless, that people will laugh at me, that I’ll feel terrible, that I’ll struggle and achieve nothing. That I won’t even be able to write. And that… that’s horrible. It’s incredibly humiliating. I could ask someone to write it for me, but I can’t. I just can’t do it. What would they think of me? That I’m illiterate? That I don’t know how?" *(F49NT1b)*

One patient described feeling deeply self-conscious when symptoms occurred in public, perceiving them as an additional burden. Another patient feared cataplexy episodes and was actively suppressing the positive emotions that cause them, leading others to mistakenly believe that he lacked a sense of humor. The patients in our sample often felt bad because of the embarrassment their family members had to experience regarding their symptom manifestation.

“For example, even when eating—when our children were small, we used to go on trips, and after the trip, we would usually go to a restaurant to eat. But since then, I’ve stopped going to restaurants because I was so embarrassed. They brought us soup, I started eating, and suddenly, I had both a cataplectic and a narcoleptic episode. Luckily, my husband was sitting next to me, or I would have fallen headfirst into my bowl. I was about to take a spoonful when the spoon slipped, splashing everything on me—so embarrassing. I ended up going outside without even finishing my soup, just to get some fresh air. Once it eased a bit, I had a cigarette outside and then came back to finish my meal. But after that, I was dozing off at the table. My husband told me never to go out to eat with him again because it was a disaster. But what can I do? How can I limit something that I have no control over when my body does it on its own?” *(F54NT1b)*

### Feelings of fear and concerns

Fear, frequently associated with the manifestations of narcolepsy, concerns about injury, or societal prejudice, emerged as a recurring theme throughout the interviews. Participants in our study expressed anxiety about potential harm resulting from cataplexy episodes. For instance, one participant feared sustaining further injuries, as she had already experienced multiple fractures and dental trauma due to sudden loss of muscle control during cataplexy. Similarly, apprehension extended to activities that might lead to physical harm or property damage. As one male patient humorously noted, he would likely no longer attempt certain activities, such as rock climbing.

Beyond the fear of immediate physical consequences, some participants also voiced concerns about disease progression. They worried about the return of symptoms they had previously managed well with treatment. Some feared excessive daytime sleepiness not only due to its disruptive nature, but also because it could leave them vulnerable to theft or harm, considering some participants have already been robbed multiple times due to their condition.

“When I walk, I’m afraid that my strength will suddenly leave me and that I will collapse somewhere. So, I sit down for a while and wait for the feeling of weakness, total exhaustion, and fatigue to pass. Sometimes, it doesn’t go away quickly, so I must wait—and heaven forbid that I fall asleep and someone robs me. [...] I fall asleep within three seconds, anywhere, which means anything could happen to me on a train.” *(F49NT1b)*

“When I started taking the medication, I stopped fainting as frequently. I’m not sure whether it was due to the medication itself or if my mental state simply improved—maybe just knowing that someone finally believed me and acknowledged that something was wrong helped me feel more at ease. I don’t know what exactly caused the improvement, but things did get better. However, I still fear that it might return and that I’ll start fainting like before. Back then, it was so severe that I couldn’t even walk from my room to the bathroom without collapsing three times along the way.” *(F26NT1b)*

In addition to the health-related fears and concerns expressed by the participants, some also reported anxiety about social isolation. They found it extremely challenging to discuss their condition and, as a result, prefer to keep it hidden from others. However, within intimate relationships, participants were more likely to disclose their condition, although they often feared a lack of acceptance from their partners. One participant noted his partner would view it as a considerable sacrifice, given that he is sometimes incapacitated by sleep for extended periods. Another male participant described how his former girlfriend was significantly disturbed by his tendency to fall asleep during their shared activities, like watching her favorite movie, etc.

“At that time, my girlfriend told me, “God, you’re sleeping all the time.” I would come home from work exhausted, lie down, and fall asleep whenever I had the chance. Even while watching TV, I would drift off. I remember she scolded me once because we had started watching a film that was very important to her, and I must have fallen asleep during it at least seventy times. She was upset and kept pointing it out. So, when I think back to the very first symptoms, what I remember most clearly is being told off for always sleeping.” *(M40NT1)*

“That kind of helplessness... I'm not even 50 years old yet, where I could be right now, and I can’t be anywhere because I must be at home... I can’t say it’s a life without meaning, because I still have my family, I have a husband, I have children, I have everything. Nothing has really changed, except that I had to reduce some things, and the worst part is that I must be at home and can’t do normal things, like cooking... I do cook, but for something bigger, someone has to be home to check if I’m cooking [not sleeping], to make sure it’s not burning... there are illnesses worse than mine, and people live with them, so I’ll live too, and as they say, pardon my expression, as long as the fat lady sings, I’ll go full speed ahead.” (*F49NT1a*)

### Self-perception and life role fulfillment

Some participants reported narcolepsy significantly impacted not only their professional roles but also their family dynamics and overall life. They expressed a sense of failure due to their inability to meet the expectations associated with various life responsibilities. Some female participants described feeling as though they were failing in their roles as wives and mothers since the onset of the disease. This perception arises from their decreased ability to dedicate time and energy to their families compared to before the condition’s development. In certain cases, participants were not able to adequately care for newborns. One participant in our study frequently experienced sleep episodes, which necessitated her mother-in-law’s assistance in caring for her infant daughter.

“But it’s more difficult to function at home, and sometimes I feel like I’m not a good enough mother, I’m not a good enough wife. This isn’t the life I imagined, and for the past six years, we haven’t really been living a normal life. We’re trying to imitate it, but we’re just in a kind of survival mode. Sometimes it would be nice to be alone, to go somewhere by myself, to take a walk, but that’s not possible, or when something needs to be taken care of, my husband always has to be there. If he can’t, someone else has to be, but there’s always this feeling that I’m dependent on someone else, that I can’t manage things on my own. Sometimes, quite often, even simple things like taking a shower. And I’m more afraid of what if I fall asleep and fall, and no one will be there, so sometimes I get stressed out about it. But I try not to think about what I can’t do, because that’s not good; it makes me more depressed; I try to think positively.” *(F28NT1)*

As her daughter grew older, she sought to engage with her mother in conversation, but her mother would often fall asleep. This ultimately impacted their relationship. Conversely, one male patient feared he might miss the birth of his own child, as he doubted his ability to handle the overwhelming joy of the situation and would experience cataplexy.

“I was worried about these things, whether I would even be able to provide support, and then I was afraid that I might end up in a situation where they would have to resuscitate me, even though I told myself that many men go to the birth, see blood, and still faint, so they probably wouldn’t even address it at all. But I know, my wife was concerned that we should probably inform them, in case something happens, so they won’t try to give me CPR and such, because with cataplexy you’re still conscious and don’t need these things. It’s not epilepsy, so the person is completely alert, just the body is shut down… or the part of the brain that controls muscle tone goes into deep sleep. She was really worried about all of this, but I have to say, everything went very well, and they even said that I was a support during the birth, and that adrenaline has a huge effect on cataplexy and narcolepsy.” *(M40NT1)*

### Coping with the burden of narcolepsy

Narcolepsy is among the disorders that profoundly impact the lives of those affected. We identified diverse ways in which patients perceive, internalize, and adapt to their condition. While some individuals have come to terms with their diagnosis, acknowledging that “it cannot be fought, only lived with,” others experience narcolepsy as an unfair and restrictive burden that prevents them from fully engaging in life.

Adjusting to a chronic, incurable disorder that significantly disrupts daily life can be a profound psychological challenge. However, for some participants, the process of acceptance played a crucial role in shaping their overall well-being. Acceptance of the disease emerged as a key factor in symptom management and coping strategies. Some individuals personified their condition, described it as a presence they had to learn to coexist with. Some participants referred to narcolepsy as to their “companion” and emphasized that while they must sometimes adapt to it, there are also moments when narcolepsy adjusts to them. One participant highlighted the futility of resisting the disorder, advocated instead for a mindset that fosters coexistence rather than conflict. The age at which narcolepsy symptoms first appear can play a crucial role in shaping the emotional experience of living with the condition.

“Yes, yes, I would try something because I have already gotten used to living with this disease. I’ve accepted it and told it, ‘You have to allow me something, and I have to allow you something.’ We respect each other—it’s like we made a deal. I take it with humor because, well, what else can I do? Cry? No, I won’t cry.” *(F54NT1b)*

Interviewer: “What emotions does having narcolepsy evoke in you?”

Patient: “Now, I don’t really care anymore because I know I have it, and there’s nothing I can do about it.”

I: “And how did you feel before you knew what it was?”

P: “I don’t know. I was still basically a child back then, so I didn’t think much about it at all. Now, I’ve come to terms with it, but at that time, if I fell asleep, I just fell asleep—I didn’t really question it. My mother was the one who was more concerned, wondering why I kept falling asleep.” *(F25NT1)*

Participants of our study reported acceptance of the disease influenced how their body responds to treatment, adherence to lifestyle modifications, and the extent to which they committed to a recommended routine. One male respondent, a professional fitness trainer, had almost eliminated episodes of cataplexy and effectively manages EDS through a strict regimen of sleep hygiene, diet, and physical activity, without the need for medication. He stated that narcolepsy has a minimal impact on his daily life, although he acknowledged that others may experience greater difficulties.

“So, definitely not letting it get to me. I might have taken it as a form of motivation, realizing that I would need to put in more effort in certain areas and, more importantly, rethink my approach—not in terms of having a problem or a diagnosis. People often see a diagnosis as a limitation, thinking, *Now I’m sick, so I can’t do this, I can’t do that.* But I looked at it differently and started thinking about what I *can* do and how I can work with it. Over time, I figured out that, essentially, I can do anything without really having to restrict myself in any way.” *(M29NT1)*

Beyond discipline and routine, an individual’s psychological approach to the condition also plays a crucial role. Patients who express less emotional burden by their diagnosis may find it easier to adapt. For instance, another male participant described himself as naturally easygoing and phlegmatic, which helped him accept narcolepsy as something beyond his control. Meanwhile, another patient developed a fascination with the vivid dreams induced by narcolepsy and actively explored the creative potential of his brain through these experiences.

“I just accept that I have these difficulties, and that’s it. I think a lot of it comes down to attitude. Even before I became a doctor—while I was still studying medicine—I always approached my condition with the mindset that this is how it is and how it will be, without thinking of it as something that defines me as a patient. For example, when dealing with professors, I never wanted to introduce myself by saying, I have this condition, so I need special treatment. Instead, I preferred that no one knew about it and tried to present myself as if I didn’t have narcolepsy at all. Of course, this approach depends on the severity of the symptoms. If someone has more severe difficulties—such as cataplexy that makes everyday tasks impossible—then hiding it isn’t an option. But for me, so far, I’ve been able to manage without letting it define me.” *(F28NT1)*

The perception of narcolepsy’s impact on daily life and the extent to which patients accept this impact significantly shaped their attitudes toward the condition. One participant experienced frequent disruptions in both their professional and personal life due to narcolepsy symptoms, leading to feelings of shame and inferiority. In contrast, another participant expresses a sense of indifference, stated they no longer care about societal perceptions, even if others viewed them as “crazy.” However, they acknowledged that for individuals who placed a high value on their public image, social reactions to their condition can be particularly distressing.

I: “What is it like to live with narcolepsy?”

P: “I don’t know what it’s like to live without it. I feel ashamed in front of people who don’t know about my condition because I don’t have a job. I feel frustrated that I can’t achieve the goals I set for myself, especially when it comes to things like diet and exercise, as I mentioned before. I feel sad that I can’t experience life to the fullest.”

I: “And what helps you cope with these feelings? When you feel sad, what makes you feel better?”

P: “I don’t think I’ve overcome these feelings… I believe I still experience them. And what could help me? It’s hard to say… I haven’t yet found a way to cheer myself up.” *(F37NT1)*

### Future aspirations and expectations of the patients

Participants who have not yet fully accepted their condition tend to express a more pessimistic outlook regarding their future. For instance, some individuals hesitate to engage in certain activities, such as travel, due to concerns about symptom exacerbation. One participant expressed a desire to go on a seaside vacation but feared that excessive excitement could trigger cataplexy, potentially leading to dangerous situations, such as drowning. Conversely, many participants reported having already achieved significant life milestones, including building families, raising children, and leading fulfilling lives. Some individuals, particularly those of older age, indicated that they no longer had major future ambitions, whereas others expressed aspirations related to independence, such as relocating abroad.

“You know, the dreams I had in life were fulfilled already. I always wanted a garden, and I got one—my husband bought it for me, which might seem trivial, but it’s meaningful. I also wanted a proper bike, and I bought it too when I was in Germany. So, those two things were important to me: the garden and the quality bike. I have a happy marriage, a very good husband, and my children turned out well. The only thing is, I’m starting to lose the energy to work because my heart is failing, so I sold the garden to my sister for a minimal, symbolic price, and I also bought a second electric bike, one for my sister to use when she comes with me to the garden. But I have everything. The only things left would be to see a wedding and to live to see my grandchildren—that’s about it. Especially the grandchildren, the little ones.” *(F54NT1b)*

Several participants articulated future goals centered on personal development, education, and professional advancement. Despite the challenges posed by narcolepsy, they remain determined to progress in life. For example, one individual expressed a strong desire to overcome excessive daytime sleepiness and secure employment in her field of study. Additionally, she aspired to pursue doctoral studies and has already identified a specific research topic of interest.

“I want to complete another course and continue doing the work I enjoy while improving my skills. I also want to lose weight, as I gained some during my hospital stays due to narcolepsy, spending a lot of time bedridden in the beginning. Now, I want to change that, and I believe it will make me feel better, both physically and mentally. Perhaps it might even improve my narcoleptic symptoms, reducing fatigue, as I suspect my current level of tiredness could be partially influenced by my weight.” *(F22NT1)*

“I still go regularly to kickboxing and boxing, actually for almost a year now. I really like it, especially because I’ve improved technically and so on. But now I realize what kind of risk I, like an idiot, was taking. If someone more trained or in better shape than me—like I am now, let’s say—had been my opponent, I could have seriously hurt myself (note: because of the arousal which may trigger cataplexy). And I think it was completely pointless, it just doesn’t make sense.” *(M40NT1)*

## Discussion

The aim of this paper was to understand how patients perceive the emotional burden of narcolepsy. We found that narcolepsy does have an emotional impact on patients suffering from this condition. This includes experiences with the disease onset, feelings of shame and embarrassment, feelings of fear and concerns, the impact on self-perception and fulfillment of important life roles, the emotional experiences of living with narcolepsy, together with the future aspirations and expectations of the participants.

The overall experience of patients with narcolepsy in our study varied from patient to patient based on multiple factors which contributed to better or worse perception of the disease’s impact. Whether or not the participants came to terms with the disease was influenced by the understanding that the disease is not life-threatening and does not progress over time. This understanding might have been influenced by the amount of time people had dealt with narcolepsy; therefore, the period of living with it most likely affected how patients experienced the emotional burden. As the experiences are influenced by multiple factors, we could also assume the older age of the participants might be an important aspect of disease acceptance. However, young patients in our study were also accepting their condition. According to Nevsimalova et al. (2009), the age at which narcolepsy manifests can influence disease severity, with younger patients being more vulnerable to severe symptoms compared to those with adult-onset narcolepsy. Therefore, understanding diagnostic delay being prolonged in the years after the first symptoms emerge or possible misdiagnoses could be crucial in the perceived emotional burden. Limited awareness of narcolepsy may also contribute to a prolonged or incorrect diagnosis, as reflected in the reported mean delay time being around 10 years (Zhang et al., 2022).

The lack of knowledge about the symptoms of narcolepsy may also contribute to the misinterpretation of symptom manifestation in front of people. Therefore, feelings of embarrassment and humiliation were also mentioned by the study participants and their families when describing the manifestation process. The knowledge gap about the disorder is unsurprising in the general population; however, Rosenberg and Kim (2015) also identified knowledge gaps among physicians and sleep specialists, which may contribute to diagnostic challenges. Addressing these gaps in knowledge is a critical factor in reducing diagnostic delays and alleviating the stress and anxiety associated with both symptoms and the diagnostic process for patients and their families, particularly considering that the highest peak of symptom severity appears to be at the time of the symptom’s onset. This period is described by patients as distressing and anxious, primarily due to a lack of information available to them and their social environment (Nevsimalova et al., 2009). Similar findings were reported by Chen et al. (2021), who observed that adolescent patients frequently associated embarrassment with EDS and cataplexy. Additionally, symptoms led to bullying at school, significantly affecting HRQoL. On the other hand, embarrassment is only one part of the experience, as patients also fear possible injuries throughout the manifestation and perceive an increase in anxiety and social isolation preventing them from experiences. Chen et al. (2021) also reported frustration when managing symptoms, irritability leading to social withdrawal, and worry about various life aspects, such as securing employment, building relationships, and maintaining independence. In our study, participants expressed concerns particularly regarding the risk of injury, symptom relapse after remission, social prejudice, and long-term life roles—including the fear of challenges in relationships and parenthood, and professional responsibilities in both work and educational settings.

Another finding of our study connected to the overall picture of emotional burden was the impact on self-perception and roles fulfillment. The patients’ perception of themselves as workers, parents, and partners contributed to their emotional experiences when being affected by the disease. Several female participants expressed feelings of inadequacy in their roles as mothers or wives due to the limitations imposed by their symptoms. They described feeling less effective in these roles than they wished, often needing support from others and struggling to meet personal or societal expectations. This was especially challenging for those whose diagnosis occurred in adulthood, as they could directly compare their ability to fulfill roles before and after the illness. To our knowledge, this specific topic has not yet been directly explored; however, it may be linked to the study participants overall perception of QoL and their tendency to compare themselves with others. As noted by Kilmartin and Day (2024), patients with narcolepsy often perceive themselves as socially and physically different from their peers and report experiencing a lack of social support. With this concept in mind, some patients might even reconsider having children due to the challenges regarding medication during pregnancy (Baker et al., 2020).

The results of our study showed that our participants in our study do not perceive emotional burden in the same way as another. This finding was supported by the fact participants talked about acceptance and its crucial role in psychological well-being, together with mindset and personality traits. Schockman et al. (2024) identified acceptance as a distinct theme in their study, categorizing patients into two groups: those who embraced their diagnosis and found relief in receiving it, and those who perceived the diagnosis negatively. The way patients interpret their diagnosis may be influenced by the stigma surrounding narcolepsy. According to Baker et al. (2020), individuals with narcolepsy are frequently subjected to stigma from teachers, co-workers, supervisors, and others, often being misperceived as lazy, careless, or even feigning their symptoms. Reducing these prejudices may be achieved through increased awareness, education, and workplace or environmental modifications tailored to the needs of individuals with narcolepsy, as suggested by Burns and Blundell (2023). Thus, the process of disease acceptance can be challenging not only on an individual level but also in social contexts. Nevertheless, acceptance appears to play a crucial role in shaping patients’ perceptions of emotional burden.

### Strengths and limitations

The qualitative approach used in this study serves as an effective complement to quantitative research, providing deeper insight into the lived experiences of individuals with serious and chronic sleep disorders such as narcolepsy. Capturing patients’ voices and first-hand experiences is essential for understanding the burden they face in daily life, particularly given the challenges of managing an incurable condition. While our research strategy may have encountered challenges related to subjectivity in coding the interviews, this limitation was mitigated by involving three independent, trained coders who employed a consensual coding approach to enhance objectivity. However, it is important to acknowledge that qualitative findings from a small sample size may have limited generalizability to the broader population of adults with narcolepsy. Also, the use of medication and treatment could have been a factor influencing the data results in this paper, 44% of the sample were medicated and several other handled the symptoms with lifestyle modifications. We see these aspects as the influencing factors affecting emotional burden and experiences, however further research could be done to find the impact of medication and lifestyle changes on emotional experience with the condition. Further research could also be performed in the matter of differences in emotional burden while comparing NT1 and NT2 patients. Nonetheless, these findings contribute valuable insights that support and complement existing research in the field.

Considering the descriptive characteristics of our sample, it is important to note the predominance of female respondents compared to males, who were underrepresented. As a result, the findings on the burden of narcolepsy and its impact on emotional experiences primarily reflect the perspectives of female participants. On the other hand, a key strength of our sample lies in the maximum heterogeneity we aimed to achieve by including patients of varying ages, education levels, employment statuses, and residential locations. Methodologically, all interviews were conducted by the first author (JH), a trained psychologist with expertise in conducting qualitative interviews, which may have influenced the depth of the interviews. Additionally, each interview was discussed with the research team throughout the data collection process to ensure methodological rigor.

## Conclusion

Looking at the results of multiple qualitative and quantitative research findings focusing on HRQoL in narcolepsy reveals notable differences in the perceived burden of the condition. As shown in a review by Hlodak et al. (2025a) some quantitative studies suggest no significant differences between individuals with narcolepsy and healthy control groups. However, the qualitative research studies highlighted a substantial perceived burden across multiple aspects of life. Our study focused on exploring the emotional burden of narcolepsy from the patients’ perspectives. The most significant emotional disturbance was reported during the initial onset of the disease, caused by the lack of information available to people and their surroundings. Throughout life, our study participants commonly experienced feelings of embarrassment, humiliation, and fear, particularly concerning their health, social interactions, and professional or personal roles. The participants strongly emphasized the importance of acceptance, as well as maintaining of future plans, expectations, and aspirations.

Our findings could benefit both patients and healthcare providers by addressing knowledge gaps, shortening diagnostic delay, and informing about more comprehensive treatment strategies. We advocate for an approach to narcolepsy treatment that extends beyond medication and lifestyle modifications aimed at symptom management. Psychological support, including interventions from psychologists, psychotherapists, and psychiatrists, may play a crucial role in disease management and overall well-being. Furthermore, our findings highlight the need for greater awareness and accommodation in schools, workplaces, and other public environments where individuals with narcolepsy may face challenges.

## Data Availability

The datasets presented in this article are not readily available because the data from this research cannot be shared due to ethical and privacy reasons related to each interviewed individual participating in the study. Requests to access the datasets should be directed to jan.hlodak@upjs.sk.
